# Brachytic2 mutation is able to counteract the main pleiotropic effects of brown midrib3 mutant in maize

**DOI:** 10.1038/s41598-022-06428-9

**Published:** 2022-02-14

**Authors:** Michela Landoni, Elena Cassani, Martina Ghidoli, Federico Colombo, Stefano Sangiorgio, Gabriella Papa, Fabrizio Adani, Roberto Pilu

**Affiliations:** 1 Dipartimento di Bioscienze, Università degli Studi di Milano, Via Celoria 26, 20133 Milan, Italy; 2 DiSAA, Genetic Laboratory, Università degli Studi di Milano, Via Celoria 2, 20133 Milan, Italy; 3 DiSAA, Gruppo Ricicla, Biomass and Bioenergy Laboratory, Università degli Studi di Milano, Via Celoria 2, 20133 Milan, Italy; 4 Dipartimento di Scienze Agrarie e Ambientali - Produzione, Territorio, Agroenergia, Università degli Studi di Milano, Via Celoria 2, 20133 Milan, Italy

**Keywords:** Genetics, Plant sciences

## Abstract

Maize is the basis of nutrition of domesticated herbivores and one of the most promising energy crops. The presence of lignin in the cell wall, tightly associated to carbohydrates, prevents the physical access of enzymes such as cellulase, limiting the carbohydrate degradability and consequently the energy value. To increase the utilization of the biomass cellulose content, the challenge of breeding programs is to lower or modify the lignin components. In maize several mutations are able to modify the lignin content and in particular the mutation in *brown midrib3* (*bm3*) gene appeared as one of the most promising in breeding programs. Unfortunately this mutation has several negative pleiotropic effects on various important agronomic traits such as stay green, lodging and susceptibility to several infections.The maize *Brachyitic 2* (*br2*) gene encodes for a putative protein involved in polar movement of auxins. br2 mutant plants are characterized by shortening of lower stalk internodes, unusual stalk strength and tolerance to wind lodging, darker leaves persisting longer in the active green state in comparison to wild type plants, suggesting a possible utilization of br2 plants to counteract the negative effects of the *bm3* mutation. In this work, we report the generation and a preliminary characterization of the double mutant bm3 br2, suggesting the potential use of this new genetic material to increase biomass cellulose utilization.

## Introduction

Maize is the main cereal used for domesticated herbivores’ nutrition and is one of the most promising carbohydrate sources for sustainable biofuel production.

The most abundant carbohydrate synthesized in plants is cellulose, which plays an exclusively structural role, being responsible for the resistance of plants to mechanical stress. Cellulose is a linear polymer of glucose units, joined by β-1,4 glycosidic linkages and organized in microfibrils by intra and intermolecular hydrogen bonds. In plants, the secondary cell wall is made of different sheets of parallel cellulose microfibrils embedded in a matrix of hemicellulose and lignin^1^.

In angiosperms, lignin polymers are assembled from three monomers, the monoignols p-coumaryl, coniferyl and sinapyl alcohols, the polymerization of which leads to the synthesis of the three lignin units: p-hydroxyphenyl (H), guaiacyl (G) and syringyl (S). The monolignols’ biosynthetic pathway requires 10 enzymes and starts with the conversion of phenylalanine into cinnamic acid by the phenylalanine ammonia lyase (PAL) enzyme, while the polymerization of monolignols into H, G and S lignin units is thought to be catalysed by peroxidases and laccases^2^.

The tight association of lignin to cellulose prevents the physical access of hydrolytic enzymes such as cellulase, and thus limits the use of cellulose as a glucose source. For this reason the challenge of breeding programs aimed at improving maize for silage and biofuel production is to lower or modify the lignin components. On the other hand, lignin plays an important role in the plant’s life, reinforcing the structural integrity of cell walls, contributing to plant standability and resistance to biotic and abiotic stresses^3^.

This antagonism between the need for a reduced lignin level to increase cell wall digestibility and the need of a sufficient lignin level to ensure plant structural integrity has suggested breeding strategies aimed at the isolation of mutants with improved cell wall digestibility with the further introgression in genetic backgrounds in which the negative effects on agronomic traits due to the increased cell wall digestibility will be significantly reduced or absent.

In maize, seven brown midrib (bm) mutants, characterized by high cell wall digestibility and brown pigmentation of the midrib have been described (reviewed by Barrière)^4^.

The mutants bm1 and bm3 resulted from mutations in genes of the monolignol pathway, respectively *CAD2* (*Cinnamyl Alcohol Dehydrogenase2*) and *COMT* (*Caffeic acid O-Methyl Transferase*) and showed a lignin reduction ranging respectively from 10 to 20% and from 25 to 40%. The mutants bm2 and bm4, which show a similar lignin reduction of about 17%, are mutations in genes acting upstream in the phenylpropanoid and monolignol pathways, respectively *MTHFR* (*Methylenetetrahydrofolate Reductase*) and *FPGS* (*Folylpolyglutamate Synthase*). The mutants bm5 and bm6, with lignin reductions of 15% and 9% respectively, are mutations in genes still unknown, while bm7 is allelic to bm1^4^.

Among the bm mutants so far characterized, bm3, showing the higest reduction of lignin content, is considered a promising genetic material for breeding programs aimed at increasing cell wall digestibility^4^. Plants with *bm3* mutation exhibit the characteristic reddish brown pigmentation of the leaf midrib starting at the four to six leaf stage and a severe reduction of S units in the lignin polymer^5^ (Table 1, by Adani et al.)^6^ as expected because the *COMT* gene, mutated in bm3 mutants, encodes for the enzyme caffeic acid o-methyl transferase, a key enzyme required for the syringyl (S) units of lignin synthesis^7^. As a consequence of the *bm3* mutation, cell wall digestibility was improved from 0.9 to 15% in comparison with isogenic non mutant hybrids^8^.

**Table 1:** Gas exchange parameters measured in the 4 AI-RILs at maturity.

	Water flow rate (cm^3^ s^−1^)	Stomatal conductance (cm s^−1^)	Transpiration rate (g cm^2^ s^−1^)
Wild type	2.95 ± 0.14^a^	0.8 ± 0.18^b^	15.35 ± 1.18^b^
br2	1.15 ± 0.36^b^	0.31 ± 0.12^c^	5.55 ± 2.08^c^
bm3	3.66 ± 0.40^a^	1.37 ± 0.26^a^	22.16 ± 3.43^a^
bm3br2	2.08 ± 0.50^b^	0.64 ± 0.20^bc^	12.43 ± 3.39^b^

Values followed by the same letter, within each column, are not significantly different (n ≥ 10, Tukey test, *p* < 0.05).

The effect of the high cell wall digestibility of the bm3 mutant was tested in studies on the performance of dairy cattle that revealed an higher intake (1.16 kg per cow per day) and an extra milk production (1.26 kg per cow per day) in dairy cows fed with bm3 silage in comparison with non mutant silage (reviewed by Barrière)^4^. The higher silage intake can be explained by the fact that forage intake depends on the time needed for chewing and ruminating, so the lower mechanical resistance and the easier degradability by rumen microorganisms of bm3 silage allows a faster ruminal passage rate, resulting in an higher intake and an higher energy value^4^.

Unfortunately the *bm3* mutation has several negative pleiotropic effects on various important agronomic characters such as reduced yield^9^ and stalk strength^10^, increased susceptibility to pathogens^11^ and to drought^12^, reduced chlorophyll content and precocious senescence^13^, that constitute important concerns for its use in feed and energy crops.

The “Green revolution” resulted in great improvements in yields thanks not only to the use of fertilizers and pesticides but also to the introduction of dwarfing mutations in rice and wheat^14^. Susceptibility to lodging is a significant issue for tall crop plants, causing significant reductions in yield and quality. Shorter varieties which are more resistant to lodging are in fact characterized by higher yields^15^.

In maize, two classes of mutants are characterized by reduced stem elongation, the dwarf mutants (dwarf plant1 (d1), dwarf plant2 (d2), dwarf plant3 (d3), dwarf plant5 (d5), dwarf plant8 (D8), dwarf plant11 (D11) and anther ear1 (an1)) and the brachytic mutants (brachytic1 (br1), brachytic2 (br2) and brevis plant1 (bv1), formerly named brachytic3 (br3)). The dwarf mutants, showing a dramatic reduction in plant height and andromonoecious ears, often associated with sterility, are impaired in gibberellin (GA) biosynthesis or signalling, while the brachitic mutants are characterized by a less severe phenotype, since they lead to the shortening of internode lengths without reduction of internode number and organs’ size and are GA insensitive^16–18^.

In particular, in br2 plants the mutation in multidrug resistant (MDR) class of P-glyoproteins (*PGPs*) gene, responsible for the polar auxin transport in maize stalks, results in shortening of the lower internodes without affecting the size or development of any other part of the plant^17^.

In a previous paper we described the isolation of a new maize brachytic mutant, *br2-23*^19^. It is characterized by a short stature due to the shortening of the lower internodes, leaves which are more erect and an increased strength of the stalk, with the girth of the second internode and vessel elements respectively 30% and 25% larger than in the wild type^19,20^. Furthermore, the *br2-23* mutation is not completely recessive, the heterozygous *Br2-237br2-23* plants showed a statistically significant reduction in plant and ear height in comparison with the wild type, suggesting a possible use of the *br2-23* allele to reduce plant height and increase standability in hybrids^21,22^.

Previously reported data suggested the good potential of brachytic-brown midrib double mutations to increase the energy value of forage. In fact, in comparison with maize, sorghum silage forage is chacterized by a lower energy value, due to lower starch and higher fiber content. This difference seemed to disappear when a brachytic brown midrib sorghum was used. The results of a trial comparing silage produced from brachytic brown midrib forage sorghum with corn silage, reported similar intake and performance for lactating dairy cows fed with the two different diets^23^.

With the aim of reducing the negative effects associated to the reduced lignin content in maize bm3 mutant we generated the double mutant br2bm3 and in this paper we present the phenotypical, molecular, histological and chemical characterization of this new genetic material.

## Materials and methods

### Plant materials

The maize (*Zea mays* L.) seed stock used in this study, as a source of *brown midrib 3–1* (*bm3-1*) mutation (W23 near-isogenic lines), was provided by the Stock Center Resources of MaizeGDB (http://www.maizegdb.org/stock.php) whilst the *brachytic 2–23* (*br2-23*) was originally isolated in the progeny of a selfed B73 inbred line plant^19^.

### Field experimentation

The maize plants were cultivated in the experimental field of the University of Milan, situated in Landriano (PV), Italy (45° 18′ N; 9° 15′ E), 88 m a.s.l. The experiment was laid out in randomized blocks. Each genotype was cultivated in three plots for a total of 12 plots. The size of each plot was about 10 m^2^ (5 m × 2.1 m), with a density of 60 plants per plot. Sufficient irrigation was provided periodically as needed to supplement rainfall. On the first DAS (days after sowing) a treatment with a pre-emergence herbicide (Clarido) was done. For fertilization, urea was utilized at the maize sixth leaf stage (200 kg/ha).

### *br2* allele genotyping

To perform cosegregation analysis, F2 and BC1 populations, obtained respectively by selfing br2-23/br2-23 B73 x+/+B73 plants and by crossing br2-23/br2-23 B73 x+/br2-23 B73 plants, were screened for brachytic phenotype and a piece of leaf was used for DNA extraction^24^. Polymerase chain reactions (PCR) were performed using BracD2 (upstream primer 5′-GCCGCGTAGGACGGA ATG-3′, position + 6645) and Brac16R (see section “cloning and sequence analysis”) primers designed on the region containing the eight-nucleotide deletion present in the 3′ region of *br2-23* allele^19^ to obtain allele-specific amplified products from *Br2* and *br2-23* alleles. The amplification fragments of 105 bp, specific for *Br2* allele, and 97 bp, specific for *br2*-23 allele, were fractionated by electrophoresis using 3% (w/v) agarose gels.

In the following sections we will refer to the br2-23 mutant as br2.

### *bm3* allele genotyping

Plant genotyping was performed using specific primers previously described^25^ and designed on the region of *bm3-1* allele containing a *B5* transposon, to obtain an allele-specific amplified product. 9 DAG (Days After Germination) seedlings were used for DNA extraction^24^. PCRs were performed using the primers PRIMER13 (reverse primer 5′-GCCCAGGCGTTGGCGTAGATG-3′, position + 1990 respect to the start codon) and PRIMER16 (forward primer 5′-CATTCAGACGTTCGCCGACTGAAGG-3′, position + 972 respect to the start codon)^25^. A specific fragment of 1019 bp was detected in the presence of the wild type *Bm3* allele, while no amplification was detected in the presence of the *bm3-1* mutant allele. The PCR products were fractionated by electrophoresis on 1% (w/v) agarose gel.

In the following sections we will refer to the bm3-1 mutant as bm3.

### Semiquantitative RT-PCR

Total RNA was extracted from wild type, br2 and bm3 single mutants and bm3br2 double mutants seedlings at the developmental stage of 12 DAG (Days After Germination) as previously described^26^. Reverse transcription polymerase chain reaction (RT-PCR) was used to detect the *Bm3* and *MZEPAL* genes transcripts. Total RNA was treated with DNAse I 1 U/μl (Deoxyribonuclease I, Amplification Grade, Invitrogen) and first strand cDNA was synthesized with an oligo (dT) primer from total RNA using the Cloned AMV First-Strand cDNA Synthesis Kit (Invitrogen). The different samples of cDNA were then diluted to obtain a uniform concentration. First-strand cDNA was used as template for PCR amplification. Amplification reactions were performed in a final volume of 50 µl containing an aliquot of cDNA synthesized from 5 µg of total RNA, 5X Green Reaction Buffer, 2.5 mM MgCl_2_, 200 µM each dATP, dCTP, dGTP, and dTTP, 0.1 µM each primer, and 1 unit of GoTaq (R) Flexy DNA Polymerase (Promega, Madison, WI). The housekeeping gene *orange pericarp-1* (*orp-1*), which encodes the β-subunit of tryptophan synthase^27^ was used to standardize the concentration of the different samples. *orp-1* specific sequences were amplified using the following primers: upstream primer, 5′-AAGGACGTGCACACCGC-3′, and downstream primer, 5′-CAGATACAGAACAACAACTC-3′. The length of the amplified product was 207 bp. Several cycles of successive cDNA dilutions and *orp-1* amplifications were done in order to obtain similar amplification signals in the different samples and to ensure that amplification reactions were within linear ranges. For *bm3* transcript amplification the specific primers used were BM21F (5′- ATCATGCACTCTGGCTGGCC-3′, position + 2181, 3′UTR) designed on the basis of the sequence of the *Zea mays O-methyltransferase* (*OMT*) gene deposited in GenBank (accession number: M73235) and PRIMER15 (5′-TCACCAAATTAAAAGAGAGCAA-3′, position + 2382, 3′UTR)^25^. For *Zea mays phenylalanine ammonia-lyase* (*MZEPAL*) gene (accession number L77912:), the specific primers used were PAL1F (5′-TAAAAGAACGCCAAGGAGAAG-3′, position + 2163, 3′UTR) and PAL1R (5′-TACTTAAACACAACAACAGTATA-3′, position + 2428, 3′UTR).

The amplified products of 202 bp for BM21F/ PRIMER15 and 266 bp for PAL1F/ PAL1R were fractionated on 1.5% (w/v) agarose gels. The identity of the products was confirmed by sequencing (cDNAs were amplified by High-Fidelity PCR, Pfu polymerase; Stratagene).

### Agronomic parameters

At maturity we measured plant height and ear height. For each genotype anlyzed at least 20 plants, randomly chosen in the three plots of the experimental field (see “Field experimentation” paragraph), were measured.

### Chlorophyll and carotenoids quantification in leaves

For each genotype analysed, mature apical leaves were collected at the flowering stage, from 10 plants randomly chosen in the three plots of the experimental field (see “Field experimentation” section), and the amount of chlorophyll (chlorophyll a and chlorophyll b) and carotenoids was quantified by spectrophotometric analysis.

Briefly, 2 ml of 80% acetone was added to 0.2 g leaf tissue grinded in liquid nitrogen (three replicated for each sample), after centrifugation the supernatant was collected and used for absorbance measurements at 645 nm (chlorophyll b), 663 nm (chlorophyll a) and at 470 nm (carotenoids).

### Histological analysis

To determine epidermal cell size, br2, bm3, bm3br2 and wild type plants, were grown in open field conditions, and at the 10 leaf stage a 2 cm^2^ square was excised from the central area of the 10th leaf and treated with a clearing solution (160 g chloral hydrate, 20 ml glycerol in 60 ml water). Cleared leaves were mounted on slides and interference contrast images were taken using a Zeiss IMAGE R.D1 microscope equipped with a AxioCam MRc1 digital camera. For each genotype, the leaves of 5 different plants were analysed and from each leaf 50–100 measurements were taken and statistically analysed.

For cell permeability and lignin analysis, br2, bm3, br2bm3 and wild type plants were grown in open field conditions and at the 3rd leaf stage a 1 cm^2^ area was excised from the central region of the 2nd leaf blade and further processed as described below. To analyse cell permeability, the leaves sampled were stained with Evan’s blue, known to enter only in cells with impaired cell wall permeability^28^. To analyse lignin content, the leaves sampled were cleared, transversal sections were hand cut with a razor blade and their natural autofluorescence (principally due to the presence of phenolic compounds such as lignin) was observed. Furthermore, to analyse more specifically the S lignin component, the hand cut leaf sections were stained with the Maule reagent (KMnO_4_) as previously reported^29^. For each genotype the leaves of 5 plants were analysed, using a Zeiss IMAGE R.D1 microscope equipped with a AxioCam MRc1 digital camera.

### Measurement of gas exchange parameters

The gas exchange parameters, water flow rate, stomatal conductance and transpiration rate, were measured using the LI-1600 Steady State Porometer.

All measurements were conducted on a total of at least 10 plants randomly chosen in the three plots of the experimental field where the four genotypes were grown in a randomized block design as previously described in “Field experimentation” paragraph. .

The measurements were carried out in open field at 12 noon in sunny weather on br2, bm3, bm3br2 and wild type plants at maturity.

### Statistical analysis

Microsoft Excel® was used to collect data, SPSS® was used to perform one-way ANOVA on sampled data. Tukey’s Test was used to analyse the difference among the 4 AI-RILs.

### Ethics approval

The experimental research and field studies on plants, including the collection of plant material, complied with relevant institutional, national, and international guidelines and legislation. The appropriate permissions and/or licenses for collection of plant or seed specimens were obtained for the study.

## Results

Generation of 4 Advanced Intercross-Recombinant Isogenic Lines (AI-RILs) (bm3br2, bm3, br2 and wild type).

**Figure 1:**
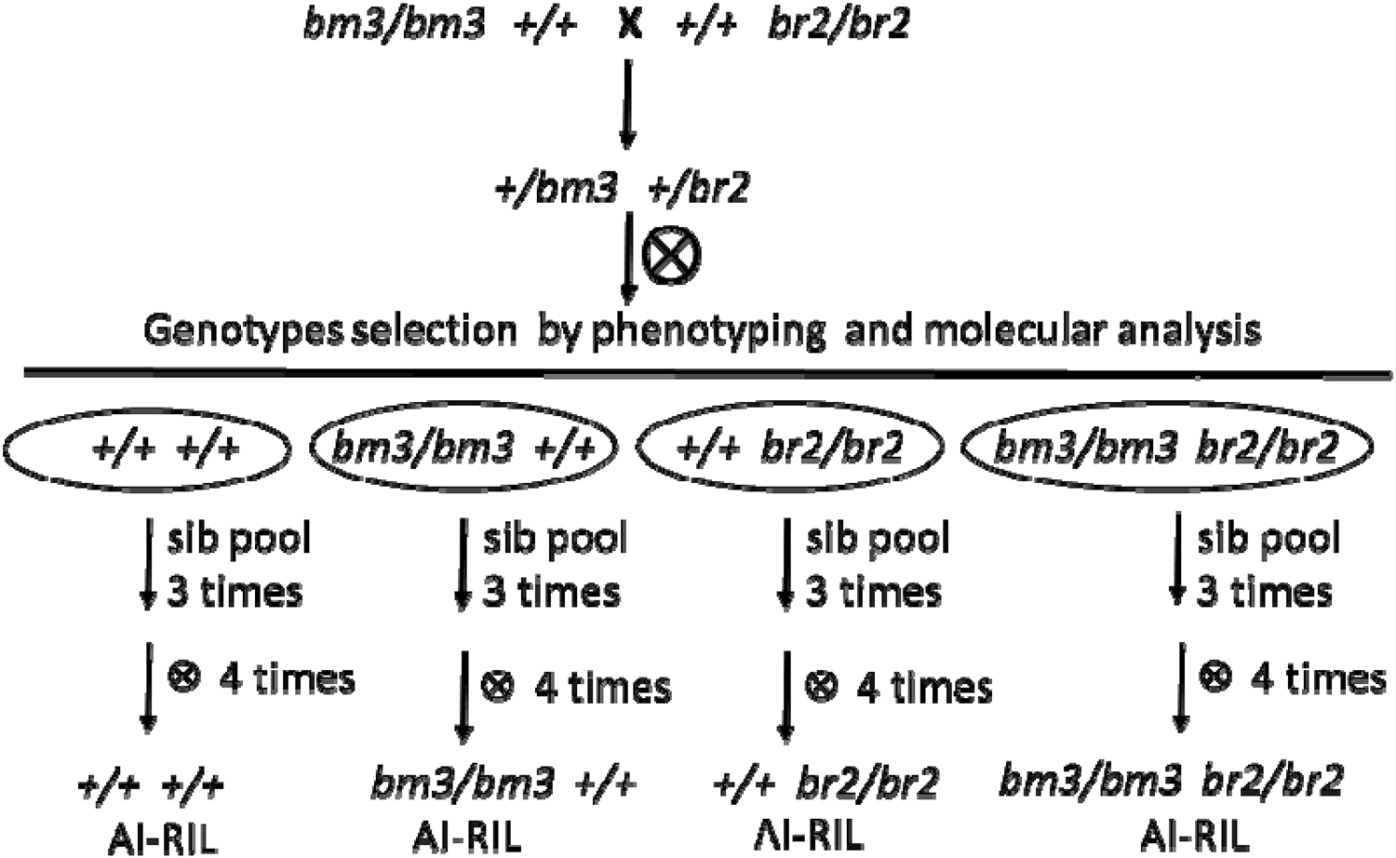
Breeding scheme for the generation of thefour AI-RILs characterized in this study.

With the aim of reducing the negative pleiotropic effects of *bm3* mutation, we crossed bm3 with br2 mutant plants. In the F2 progeny we isolated the 4 genotypes: the wild type control (++), the *brachytic 2* (*br2*+*,* the *brown midrib 3 *(+*bm3*) and the double mutation *bm3br2*. The pedigree selections were assisted by molecular genotyping of the plants using primers specific for *br2/Br2* and *bm3/B3* alleles. 5 to 10 F2 plants were selected for each genotype and crossed with each other by pollen pooling for three generations and after 3 more cycles of self pollination we obtained the the 4 AI-RILs, the characterization of which is the object of this paper (Fig. 1).

### Phenotypic characterization of the 4 AI-RILs

**Figure 2:**
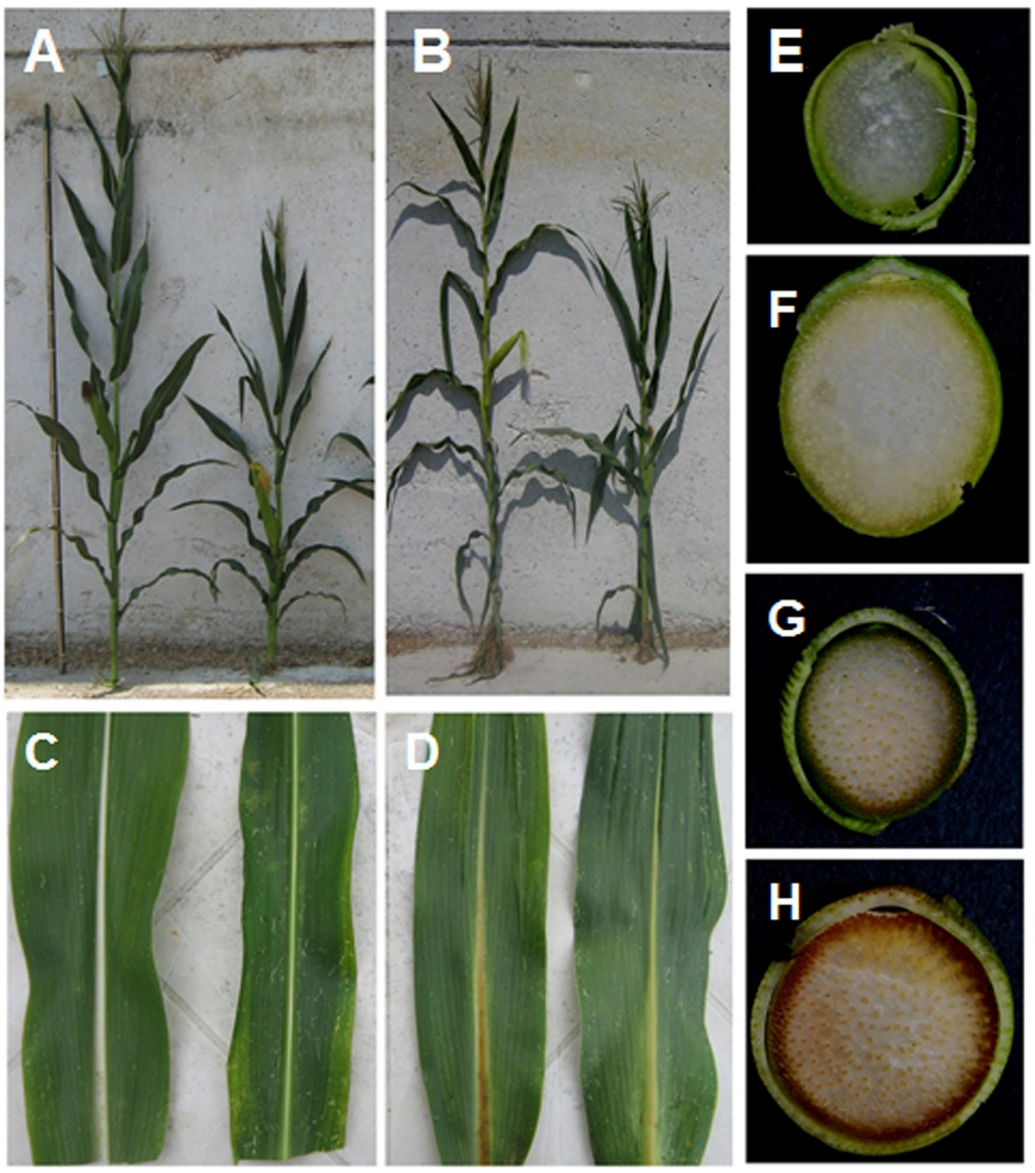
Phenotype of the 4 AI-RILs at maturity. Whole plants: (**A**) wild type (left) and br2 (right), (**B**) bm3 (left) and double mutant bm3br2 (right). Phenotype of adaxial (left) and abaxial (right) leaf side: (**C**) wt and (**D**) bm3. First internode stalk section: (**E**) wild type, (**F**) br2, (**G**) bm3 and (**H**) bm3br2 plants.

We sowed the 4 AI-RILs in an open field to compare their phenotypes at maturity. In particular we analyzed plant shape, leaf angle, midrib area, and culm diameter (Fig. 2).

The br2 mutant at maturity was characterized, as previously reported^19^ by short stature, compact lower internodes, broader, darker and erect leaves with increased midrib area and stalk section significantly larger in comparison with the wild type (Fig. 2A,F). The bm3 plant height appeared not significantly different in comparison with the the wild type (Fig. 2A,B), the leaves were characterized by brown midrib (Fig. 2D) and the stalk section, of which the diameter was comparable to that of the wild type, showed a brown pigmentation at the periphery of the central cylinder (Fig. 2E,G). The double mutant bm3br2 showed a combination of bm3 and br2 phenotypical characteristics: short stature, larger stalk section, broader and erect leaves with increased midrib area as in the single mutant br2*,* brown pigmentation in leaf midrib and in stalk section as in the bm3 mutant (Fig. 2B,H).

### Expression analysis

**Figure 3:**
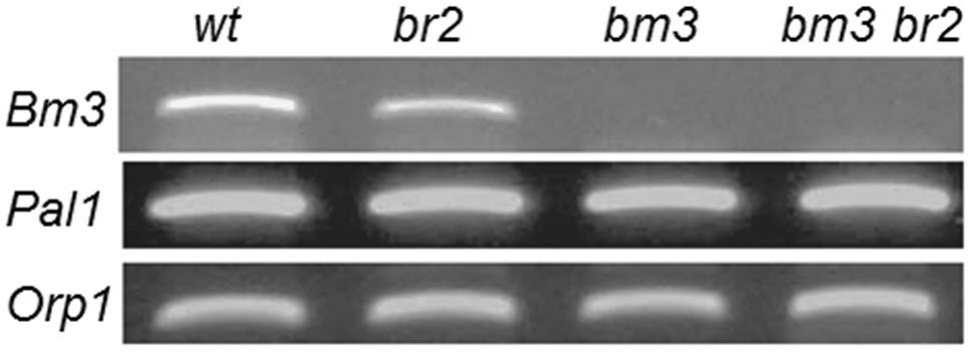
RT-PCR expression analysis of *Bm3* and *Pal1* genes*. Orp 1* gene was used as control. The RNA was extracted from seedlings of wild type, br2, bm3 and bm3br2 double mutants. The *COMT* (*Caffeic acid O-Methyl Transferase*) gene, of which the mutation causes the bm3 phenotype was indicated as *Bm3*.

We checked the effect of the *bm3* mutation on the expression level of the *COMT* (*Caffeic acid O-Methyl Transferase*) gene, the mutation of which causes the bm3 phenotype, and *Pal1* gene, catalyzing the first step of lignin biosynthesis, looking for a possible feed-back effect of the *bm3* mutation on the whole lignin biosynthetic pathway. Semi-quantitative RT PCR analysis showed no *Bm3* transcript in bm3 and in bm3br2 mutants while in br2 mutants the amount of *Bm3* transcript was slightly lower than in wild type (Fig. 3 and Supplementary Fig. S1 online).

The expression level of *Pal1* gene showed no differences among the 4 NILs analysed (Fig. 3).

### Agronomic traits analysis in the 4 AI-RILs

To characterize from the agronomic point of view the 4 AI-RILs, we measured, at maturity, some agronomic traits, in particular plant and ear height, chlorophyll and carotenoid content.

**Figure 4:**
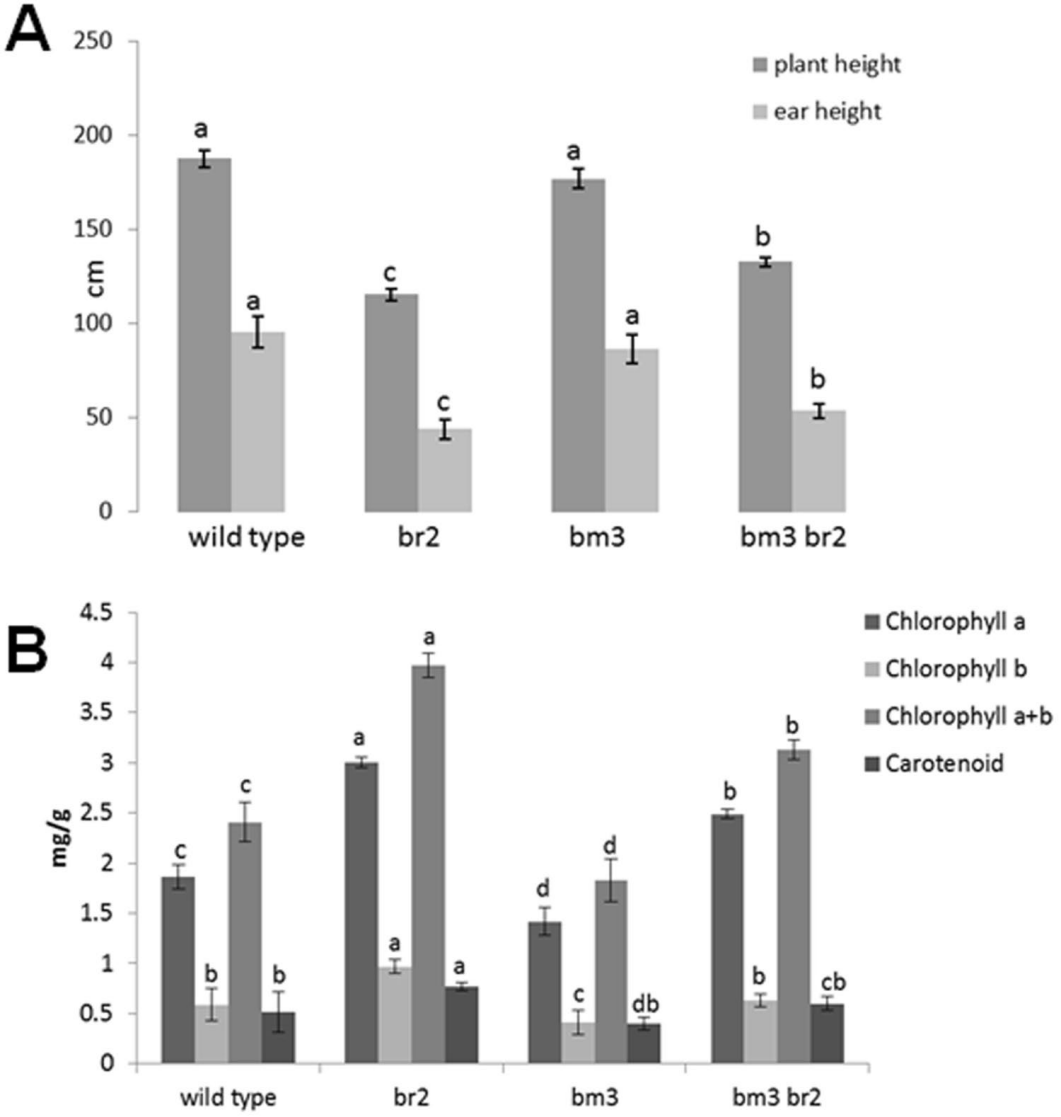
Agronomical tratits of the four AI-RILs. (**A**) Plant and ear height at maturity. SD are shown (n ≥ 20) (**B**) Chlorophyll and carotenoid content in the leaf at the silage stage. SD are shown (n = 10). For each parameter measured different letters indicate statistically significant differences (Tukey test, *p* < 0.05).

Concerning plant and ear height, no differences were found between wild type and bm3 plants, while br2 and bm3br2 plants showed a statistically significant reduction in both the parameters measured in comparison with the wild type (Fig. 4A).

The quantification of chlorophyll a, chlorophyll b and chlorophyll a + b showed, in comparison with the wild type, a statistically sigificant increased level in br2 and a decrease in bm3 mutants, while a level intermediate between br2 and bm3 was found in bm3br2 double mutants (Fig. 4B).

### Response of the 4 AI-RILs to drought stress

**Figure 5:**
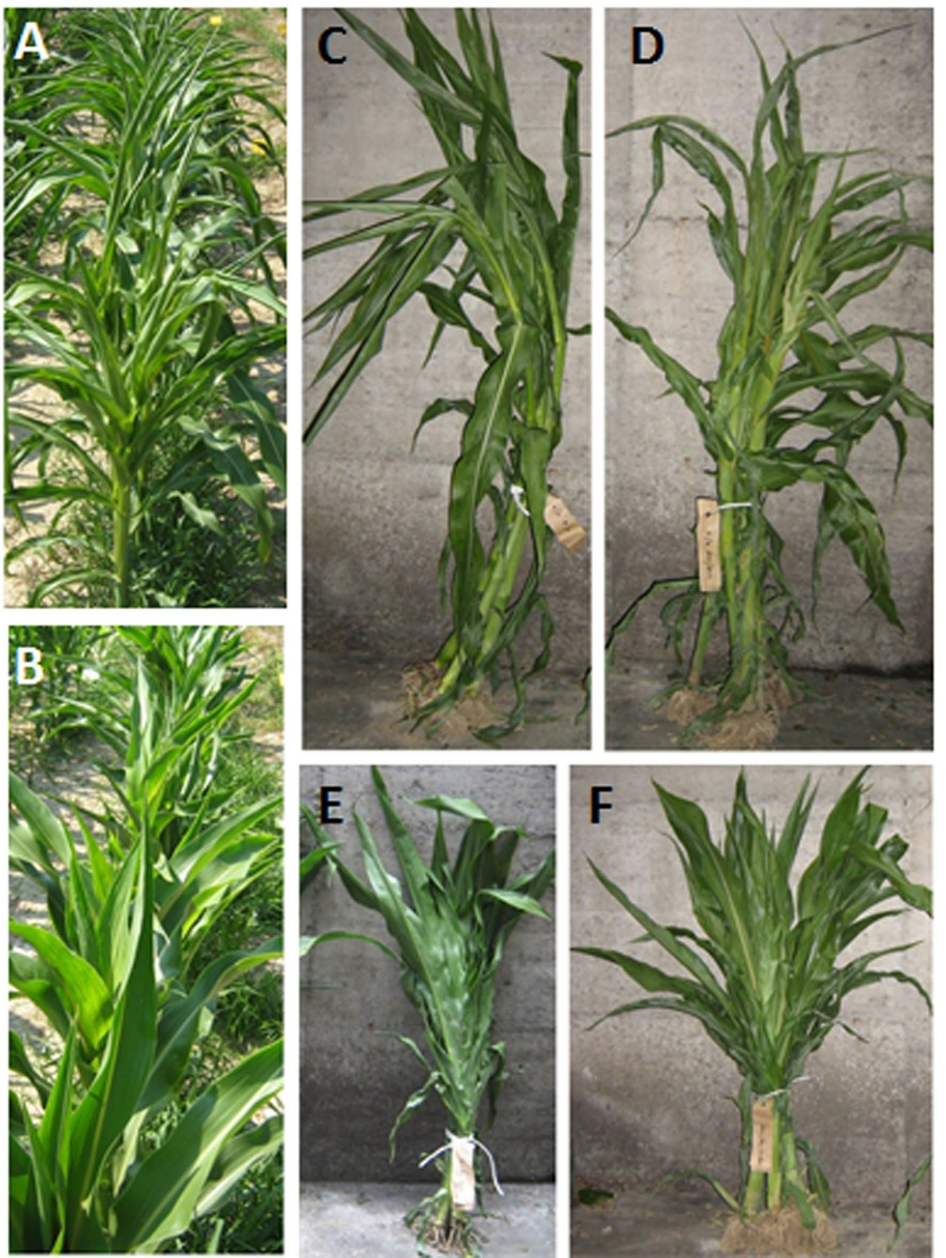
Phenotype of plants at the anthesis stage under moderate drought conditions in open field. (**A**) bm3 and (**B**) bm3br2. Phenotype of rootless plants at the anthesis stage, 2 h after the harvest. (**C**) wild type, (**D**) bm3, (**E**) br2 and (**F**) bm3br2.

bm3 plants are reported to be particularly sensitive to drought stress, while br2 mutants have been reported to have erect and darker leaves that persist longer in the active green state in comparison with the wild type, so we compare the resistance to drought stress of bm3 and bm3br2 mutants grown in open field conditions. In moderate drought stress conditions, bm3 adult plants appeared clearly wilted while the double mutants appeared healthy with no evident signs of suffering from water deprivation (Fig. 5A,B).

Because the withering of bm3 plants could be due to increased water loss from roots or from leaves, to test that hypothesis we cut wild type, bm3, br2 and bm3br2 adult plants 5 cm above the soil and allowed them to dry in the shade. In this way we could specifically check the role of water loss from the leaves in bm3 withering.

Two hours after the harvest, wild type plants showed signs of withering (Fig. 5C), that were more evident in bm3 mutants (Fig. 5D), while br2 and the double mutants bm3br2 (Fig. 5E,F) appeared not stressed, darker, more erect and turgid in comparison with the wild type.

For a better characterization of the different responses of bm3 and bm3br2 mutants to drought stress we measured in the 4 AI-RILs water flow rate, stomatal conductance and transpiration rate.

The mutant bm3 was characterized by a serious loss of water from the leaves, as shown by the high values of water flow rate (3.66 cm^3^ s^−1^), stomatal conductance (1.37 cm s^−1^) and transpiration rate (22.16 µg cm^2^ s^−1^) (Table 1). However, in the mutant br2 the loss of water was significantly reduced compared to bm3 and to the wild type phenotype: the water flow rate recorded was 1.15 cm^3^ s^−1^, while stomatal conductance and transpiration rates were lower at 0.31 cm s^−1^ and 5.55 µg cm^2^ s^−1^ respectively. An intermediate situation occurred in the double mutant: the introgression of *br2* mutation into the *bm3* backgroud drastically reduced the loss of water. In fact, all the three parameters measured in the bm3br2 mutant were halved compared to the bm3 single mutant (Table 1).

### Histological analysis

**Figure 6:**
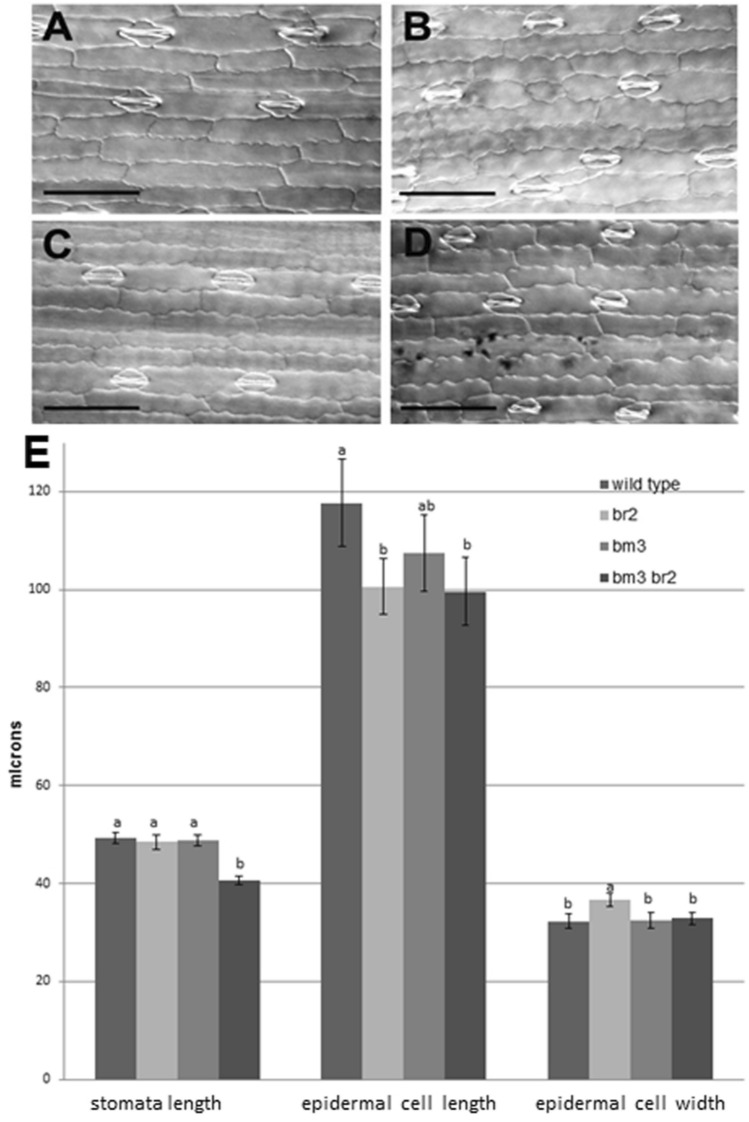
Histological analysis of the four AI-RILs. Abaxial leaf epidermidal cells of (**A**) wild type, (**B**) br2, (**C**) bm3 and (**D**) bm3br2. Bars = 100 μm. (**E**) Measurements at maturity of stomata cells length and leaf epidermal cell length and width. SD are shown (n > 50). For each parameter measured different letters indicate statistically significant differences (Tukey test, *p* < 0.05).

We analysed the effect of *bm3* and *br2* mutations on cells’ dimension in single and double mutants in comparison with the wild type. The double mutants bm3br2 showed a statistically significant reduction of stomata and epidermal cell length in comparison with the wild type, while cell width was unaffected (Fig. 6D,E).

The single mutant bm3 showed no statistically significant difference in comparison with the wild type for all the parameters measured (Fig. 6C,E), while br2 epidermal cells showed statistically significant reductions in length and increases in width (Fig. 6B,E). No difference in stomata cell number per unit area was observed when comparing the 4 AI-RILs (data not shown).

**Figure 7:**
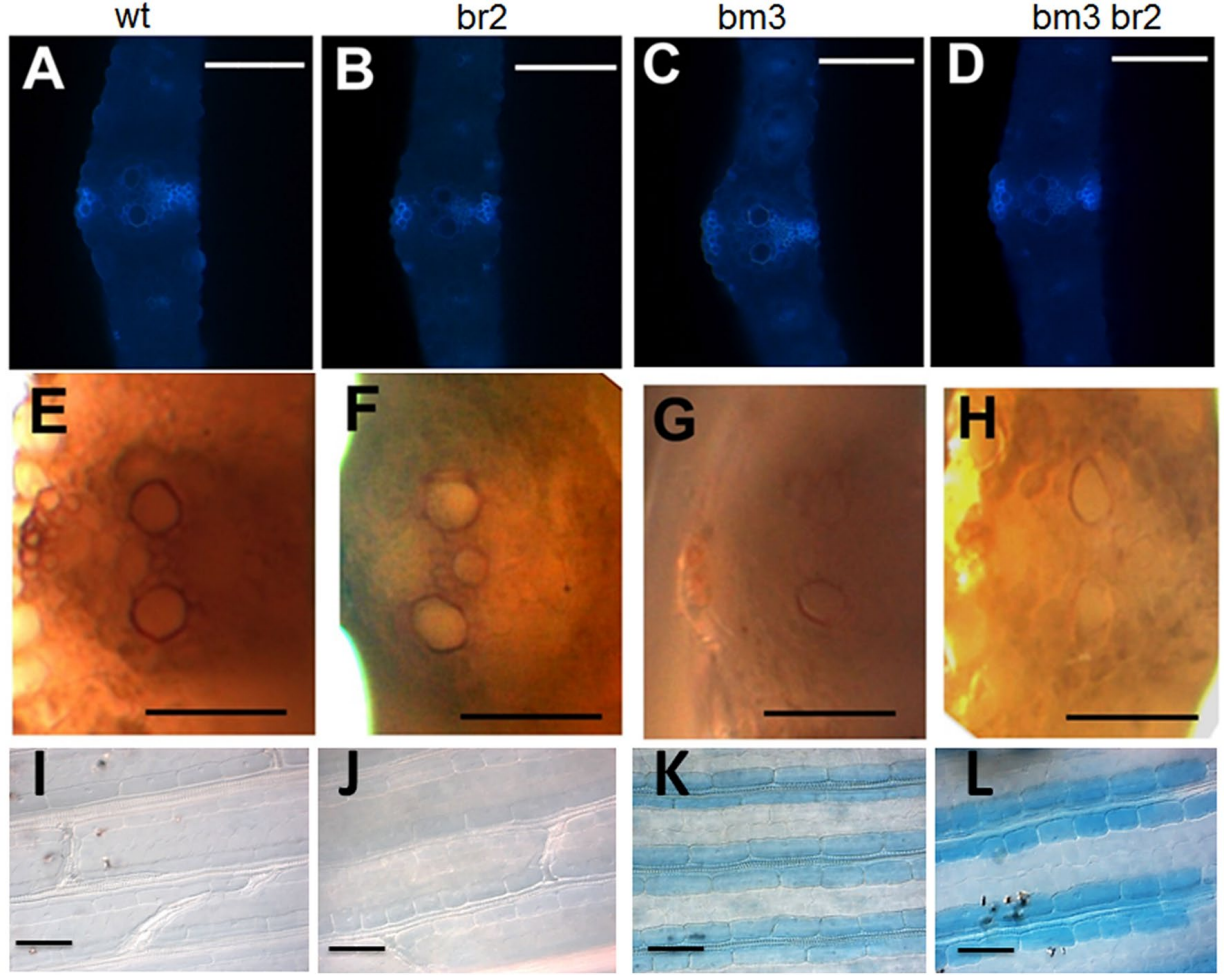
Histochemical analysis of the four AI-RILs. Autofluorescence of lignin in the seedling leaf vessel element. (**A**) wild type, (**B**) br2, (**C**) bm3 and (**D**) bm3br2, Bars = 100 µm. Maule reagent staining of the S lignin component: (**E**) wild type, (**F**) br2, (**G**) bm3 and (**H**) bm3br2. Bars: 50 µm. Evan’s blue staining: (**I**) wild type, (**J**) br2, (**K**) bm3, (**L**) bm3br2. Bars: 100 μm.

To analyse for the presence of phenolic compounds, among which lignin was the most represented, we analysed the leaf natural autofluorescence on transversal hand cut sections. No differences were found on comparing the autofluorescence detected in correspondence to the vascular elements in br2, bm3, br2bm3 and in wild type leaves (Fig. 7A–D).

To analyse more specifically the S lignin component, the hand cut leaf sections were stained with the Maule reagent. A clear decrease in the red staining visible in correspondence to vascular elements was observed in bm3 and bm3br2 mutants in comparison with the wild type, while the staining shown by the br2 mutant was similar to the control (Fig. 7E–H). To analyse cell permeability we performed the Evan’s blue staining that specifically stains the cells of which the cell wall, because of the loss of integrity and impermeability, allows the loading of the blue stain in the cell. No signal was detected in wild type and br2 mutant leaves (Fig. 7I,J) while both bm3 and bm3br2 mutants were characterized by an intense blue staining in the cells surrounding the vessels, the staining being more intense in the double mutant (Fig. 7K,L).

## Discussion

The plant cell wall is a complex structure playing a fundamental role in many different processes such as plant morphogenesis, mechanical support to the plant body, water and nutrient transport, biotic and abiotic stress response^3^.

Cell wall biosynthesis starts with the formation of the primary cell wall, deposited during cell elongation and composed of cellulose, pectins and xylans, then, when cell elongation stops, the secondary cell wall is assembled with lignin deposition, increasing the cell wall thickness, rigidity and impermeability^30^.

Cellulose is the most important energy source in crops used as forage, for ruminant animals; and biomass, for anaerobic digestors. However the accessibility to cellulose, needed to allow its conversion in free glucose by cellulases, is limited by its being tightly linked to lignin.

For this reason, to increase the crop’s energy value, breeding strategies are aimed at the isolation of mutants with improved cell wall digestibility through a reduction/modification of lignin. To counteract the negative effects of lignin reduction/modification on plant structure, the further step will be the introgression of lignin reduction in backgrounds in which the negative effects on agronomic traits due to the increased cell wall digestibility will be significantly reduced or absent.

The maize brown midrib mutants (bm) are a well studied class of mutants characterized by brown pigmentation of the midrib and high cell wall digestibility due to alterations in the lignin biosynthetic pathway^4^. The very attractive trait associated with *bm* mutations is an increased energy value when these mutant lines, isolated in different species such as sorghum, pearl millet and rice, are used to produce feed or biofuel.

Among the maize bm mutants so far characterized, the bm3 mutant showed the highest reduction of lignin content and is considered one of the most promising genetic materials for breeding programs. However bm phenotypes are also linked to some negative traits such as reduced stalk strength, increased pathogen susceptibility and precocious senescence^4,13^.

The *Brachytic 2 gene* (*Br2*) encodes for a putative protein (Multidrug Resistant class of P-glycoproteins) involved in polar movement of auxins^17^. The phenotypic traits of the br2 mutant, i.e. short stature, increased strength of the stalk and stay green phenotype, suggested the possibility of using this mutation to tackle the negative pleiotropic effects of the *bm3* mutation^19^. For this reason we crossed bm3 and br2 mutants and by marker assisted selection we isolated 4 AI-RILs: wild type, bm3 and br2 single mutants and bm3br2 double mutant (Fig. 1).

Expression analysis confirmed the absence of the wild type *Bm3* transcript in bm3 single mutant and in bm3br2 double mutant and suggested the absence of a feedback effect of *bm3* mutation on *Pal1,* the gene catalyzing the first step of the phenylpropanoid/flavonoid pathway (Fig. 3).

Phenotypic analysis on adult plants showed that the bm3br2 mutant displayed typical traits of both parents, reduced elongation and increased internode diameter like the br2 mutant, brown pigment accumulation in stalk section like the bm3 mutant (Figs. 2, 4A).

Stalk strength is an important agronomic trait, strong stalks contribute to lodging-resistence that directly impacts the yield. In a recent paper QTL mapping analysis showed that the rind penetrometer resistance (RPR), a parameter used to evaluate stalk strength, is a quantitative trait with high genetic complexity, controlled by multiple genes with minor effects and the candidate genes are involved in the regulation and formation of cell wall components^31^.

Measurements of chlorophyll content and analysis of drought stress response in the 4 AI-RILs showed that the presence of the *br2* mutation in the *bm3* background is able to counteract the precocious senescence and the high susceptibility to drought stress characterizing the bm3 phenotype (Figs. 4B, 5F). A premature onset of senescence has been reported for the rice brown midrib leaf (bml) mutant, characterized by a reduced content of chlorophyll, upregulation of senescence induced and senescence related genes and downregulation of photosynthesis related genes^13^. Our results showed that in the double mutant bm3br2, the high chlorophyll content and the stay green phenotype linked to the *br2* mutation appeared to compensate the negative effects of the *bm3* mutation, increasing the chlorophyll content and delaying the onset of senescence.

To study the drought stress response, the four AI-RILs were grown in the open field in moderate water scarcity conditions and we observed that while bm3 plants showed clear signs of drought stress, due to an increase in water loss from the leaves, the drought stress symptoms were not visible in bm3br2 plants (Fig. 5). Measurement of gas exchange parameters confirmed the higher level of water loss in the bm3 mutant, with the br2 mutant showing a lower level and the double mutant was characterized by an intermediate water loss between bm3 and br2 (Table 1). Literature data showed that in the internode of sorghum plants mutated in *df3/shabcb1* gene, orthologous to *Br2*, there is a 3–4 times higher level of peroxidases, involved in lignin and suberin synthesis^32,33^. Thus a higher level of lignin/suberin, resulting in a higher membrane impermeability, could explain the data showing a reduced water flow and transpiration rate in br2, not only in comparison with bm3 and bm3br2 mutants but also in comparison with the wild type (Table 1).

To check whether differences in cell wall composition affecting cell permeability could contribute to the observed difference in drought resistance, we analysed lignin content/composition in the different AI-RILs by histochemical analysis. As shown in Fig. 7, while there are no obvious differences in the total amount of lignin in the four genotypes studied in this work, there is a remarkable reduction of the S lignin component in the presence of the *bm3* mutation, in comparison with the wild type, as previously reported by gas chromatography/mass spectrometry analysis. In this study the S/G ratio, a measure of the crosslinks between lignin and other cell wall components, and therefore of cell wall degradability, is reported to be higher in wild type than in the bm3 mutant^6^. Furthermore, because it is known that the S/G ratio is positively linked to cell wall impermeability, with higher S/G ratio determining higher impermeability^6^, we analyzed the membrane impermeability in the four AI-RILs through Evan’s blue staining. The blue colour observed in bm3 and br2bm3 bundle sheath cells (Fig. 7K,L) highlighted the impairment of cell wall impermeability in these mutants and supported the hypothesis that the *bm3* mutation, causing alteration in cell walls with a reduction of the S/G ratio in lignin composition, resulted in increased cell permeability.

The sensitivity of bm3 plants to drought stress seemed not to be associated with a change in leaf epidermal cells’ size and shape, but some significant differences were observed among the 4 AI-RILs (Fig. 6). In particular br2bm3 stomata length appeared significantly smaller in comparison with wild type and the single mutants (Fig. 6E), while no difference in stomata density was observed.

It has been reported that stomatal size and density determine the value of stomatal conductance^34^, suggesting the hypothesis that the morphological effect of the *br2* mutation in the *bm3* background, determining a reduction in stomatal size without affecting stomatal density, resulted in a recovery of values of stomata conductance and transpiration which in the bm3br2 mutants were not statistically different from the wild type (Table 1).

Taking together these results, we can hypothesize that the observed increased tolerance to water stress of the double mutant bm3br2 is the consequence of the reduced stomatal size shown by bm3br2 leaf epidermal cells (Fig. 6) that compensate for the high cell permeability caused by the bm3 mutation (Fig. 7, Table 1) allowing a reduction in water loss (Fig. 5) and a better response to drought stress (Fig. 5).

## Conclusion

This work is the first report on the study of the double mutant br2 bm3. The presence of the *br2* mutation is able to compensate for some negative pleiotropic effects of the *bm3* mutation such as precocious senescence and low drought tolerance. This new genetic material could be a useful starting point to increase the energy value of maize varieties used as silage for animal nutrition and biomass for green-energy.

## Supplementary Information

Supplementary Information.
