# Supplementary material for: Brachytic2 mutation is able to counteract the main pleiotropic effects of brown midrib3 mutant in maize
Source: Sci Rep. 2022 Feb 14;12:2446. doi: 10.1038/s41598-022-06428-9 (PMC8844417; doi:10.1038/s41598-022-06428-9)
Supplement: Supplementary file 1 — Supplementary Information. [file 41598_2022_6428_MOESM1_ESM.pdf]

RT-PCR expression analysis of Bm3, Pal1 and Orp 1 genes.

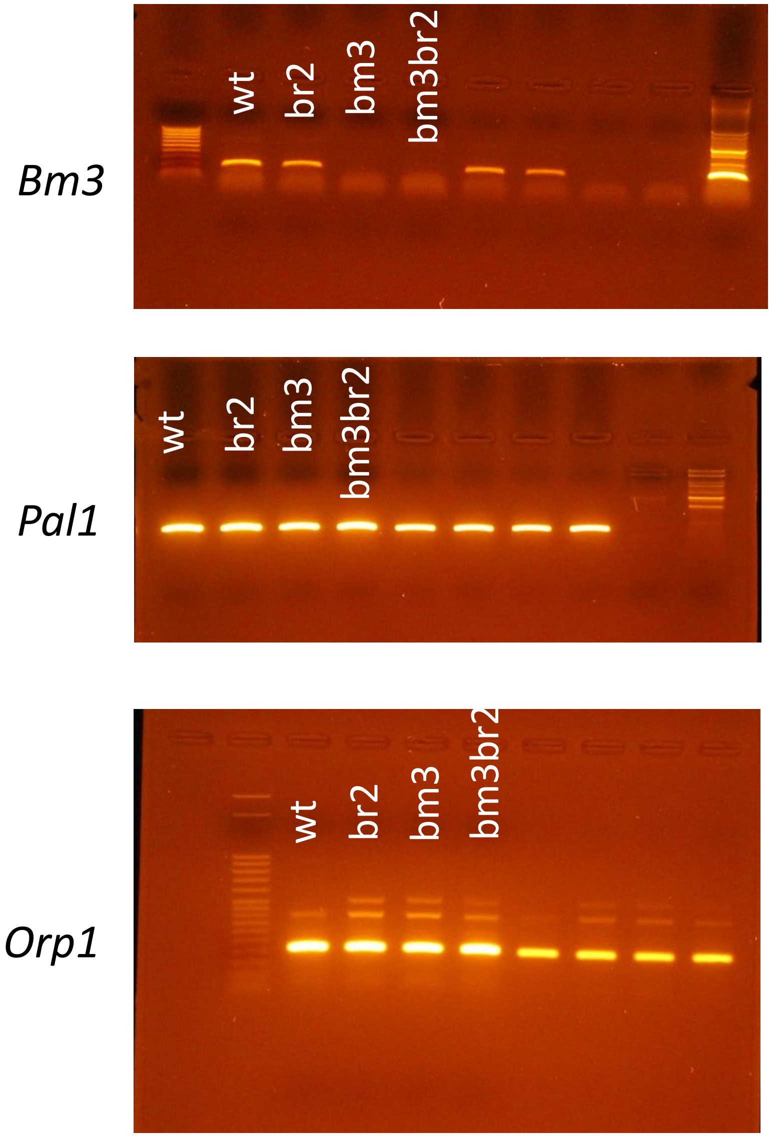

Figure S1: Original gels of RT-PCR expression analysis of *Bm3*, *Pal1* and *Orp* 1 genes. The RNA was extracted from seedlings of wild type, br2, bm3 and bm3br2 double mutants.
